# Effects of Dust and Moisture Surface Contaminants on Automotive Radar Sensor Frequencies

**DOI:** 10.3390/s25072192

**Published:** 2025-03-30

**Authors:** Jeongmin Kang, Oskar Hamidi, Karl Vanäs, Tobias Eidevåg, Emil Nilsson, Ross Friel

**Affiliations:** 1 School of Information Technology, Halmstad University, 30118 Halmstad, Sweden; emil.nilsson@hh.se (E.N.); ross.friel@hh.se (R.F.); 2 Volvo Car Corporation, 41878 Gothenburg, Sweden; oskar.hamidi@volvocars.com (O.H.); karl.vanas@volvocars.com (K.V.); tobias.eidevag@volvocars.com (T.E.)

**Keywords:** autonomous vehicles, radar, surface contamination, object detection

## Abstract

Perception and sensing of the surrounding environment are crucial for ensuring the safety of autonomous driving systems. A key issue is securing sensor reliability from sensors mounted on the vehicle and obtaining accurate raw data. Surface contamination in front of a sensor typically occurs due to adverse weather conditions or particulate matter on the road, which can degrade system reliability depending on sensor placement and surrounding bodywork geometry. Moreover, the moisture content of dust contaminants can cause surface adherence, making it more likely to persist on a vertical sensor surface compared to moisture only. In this work, a 76–81 GHz radar sensor, a 72–82 GHz automotive radome tester, a 60–90 GHz vector network analyzer system, and a 76–81 GHz radar target simulator setup were used in combination with a representative polypropylene plate that was purposefully contaminated with a varying range of water and ISO standard dust combinations; this was used to determine any signal attenuation and subsequent impact on target detection. The results show that the water content in dust contaminants significantly affects radar signal transmission and object detection performance, with higher water content levels causing increased signal attenuation, impacting detection capability across all tested scenarios.

## 1. Introduction

As the automotive industry advances towards fully autonomous driving (AD) and more sophisticated Advanced Driver Assistance Systems (ADASs), the role of vehicle perception systems becomes increasingly critical [1,2,3,4]. Perception and sensing technologies—particularly radar sensors—are essential for these systems to function effectively and reliably. Radar sensors provide robust detection capabilities, including distance, velocity, and angle measurements, which are crucial for detecting and tracking other vehicles, pedestrians, and objects in real time under various driving conditions [5,6]. However, the performance of automotive radar sensors can be compromised by surface contaminants on the sensor/radome, such as dirt, water, temperature, and other environmental debris, which can alter the radar signal and impair sensor accuracy and functionality [7].

Automotive radar (since 2022) operates within the frequency range of 76–81 GHz. However, contamination of the sensor surface introduces various signal interference issues, such as attenuation and polarization effects, which can degrade radar accuracy. Contaminants can attenuate the radar signal, distort the return signal profile, and alter polarization, all impairing the sensor’s ability to detect and interpret objects accurately [8,9,10]. Consequently, understanding the impact of these contaminants on radar signal integrity is vital for the development of reliable sensor placement and protection strategies, especially given the prevalence of adverse environmental conditions encountered in real-world driving scenarios [11,12].

Several studies have presented work on the susceptibility of radar and other automotive sensors to environmental factors. For instance, research conducted by the authors of [13] highlights that the presence of moisture on the radar surface can lead to significant signal loss, compromising radar performance. These effects are not solely dependent on the type of contaminant but also on the configuration and positioning of the radar sensor [14]. Improper sensor placement can exacerbate contamination issues, as certain vehicle areas are more prone to the accumulation of debris due to aerodynamics and surface design [15]. Snow accumulation on sensor surfaces can lead to severe contamination, and a study has presented experiments and numerical modeling of snow deposition on vehicle bodies, showing that accumulation depends on particle and the aerodynamic characteristics [16]. Additionally, automotive radomes, designed to protect radar sensors from the environment, can accumulate contaminants, thereby diminishing their intended protective role and introducing further signal distortion [17].

Recent advancements in automotive radar technology aim to enhance sensor resilience against environmental influences. Techniques such as hydrophobic coatings, heating elements, and sensor-cleaning systems have been proposed as potential solutions to mitigate the effects of contaminants. While these approaches have shown promise, they also come with challenges in terms of cost, maintenance, and effectiveness over prolonged use. Moreover, there is a growing interest in the use of aerodynamic design to deter contaminant adhesion naturally, as well as radar algorithms that can adapt to varying signal conditions. These new efforts and existing solutions would benefit from more information from systematic studies on the quantifiable effects of surface contamination on raw radar signals.

Studies have actively been presented to analyze the quantitative effects of sensor surface contamination and blockage. These studies have examined the signal attenuation of radar caused by contamination, including water, saltwater, ice, and sand, in a controlled laboratory environment [18,19,20,21]. However, these studies lack an analysis of the effect of contamination on target detection at the signal level. To assess target detection performance, studies have examined the degradation caused by contamination of the sensor cover. Various contaminants, including dust, water, oil, and dew, were applied to the cover, and their effects on sensor performance were quantified [22,23,24]. Additionally, research has explored the impact of sensor blockage on target detection and the definition of surface contamination on sensors mounted on real vehicles, with the goal of detecting multiple targets [25]. However, these studies focus primarily on light detection and ranging sensors, and the analysis of surface contamination’s effect on radar target detection remains limited. In addition, research on the impact of contaminants caused by standardized dust and water and their combinations on the sensor surface on radar sensor performance remains insufficient. Therefore, continued research is needed to understand the effects of quantified, standardized dust and water mixtures on radar signal attenuation and target detection.

To address these challenges and research gaps, this paper proposes a systematic investigation of the quantified effects of surface contaminants, such as moisture and standardized dust, on automotive radar performance in a controlled laboratory environment at room temperature. This study leverages a 76–81 GHz radar, a 72–82 GHz automotive radome tester, a 60–90 GHz vector network analyzer system, and a 76–81 GHz radar target simulator to provide quantitative insights into the influence of contaminants on the characteristics of the radar signal, including signal attenuation, and their subsequent effect on target detection. A representative 4 mm thick polypropylene plate was deliberately contaminated with controlled variations in moisture and standardized dust to simulate contaminant conditions and analyze the response of the radar signal. The contributions of this paper are summarized as follows:

- We propose a quantitative measurement method for surface contamination caused by standardized dust and moisture combinations, utilizing a variety of analytical systems, including a 76–81 GHz radar, a 72–82 GHz automotive radome tester, and a 60–90 GHz vector network analyzer;
- The impact of surface contamination on radar signal characteristics, including signal attenuation, is quantitatively measured and analyzed, with an emphasis on the effects of moisture and standardized dust combinations;
- We demonstrate the impact of surface contamination on radar signal attenuation and its subsequent effect on target detection performance, with a focus on the quantitative measurement and analysis of how contaminants influence a radar’s ability to detect objects in a controlled environment.

## 2. Methods and Measurement Setup

### 2.1. Analytical Approach

The radar equation relates received radar signal power $P_{r}$ to transmitted radar signal power $P_{t}$; it is defined as follows:

(1) $$P_{r}=\frac{P_{t}\lambda^{2}G_{t}G_{r}\sigma }{{\left(4\pi \right)}^{3}R^{4}},$$

where λ is the signal wavelength, $G_{t}=G_{r}$ are the transmitter and receiver antenna gain, respectively, *R* is the distance between the radar sensor and the target with radar cross-section σ.

The radar sensor sensitivity can be described as a noise-equivalent radar cross-section $\sigma_{noise}$ at a given distance $R_{0}$. If the radar signal deteriorates due to the soiling of the car exterior, leading to an increased excessive attenuation *L*, the noise equivalent radar cross-section will increase to $\sigma_{L}=\sigma_{noise}L$ for the distance $R_{0}$. Thus, soiling of a car can cause weak targets, such as pedestrians, to evade detection or be detected at a shorter distance. With an excessive attenuation of L=−12 dB, the detection range is halved.

Radar transmitting and receiving antennas are commonly placed near each other on a car. They will, accordingly, experience the same contamination and soiling, leading to the conclusion that *L* is the result of a two-way transmission through the contaminating material. A one-way signal attenuation of −6 dB results in halving the detection range for the radar. It is expected that even modest water content ratios in the contaminating material will cause significant signal loss. The dynamic process of gradually drying a moist material is of special interest.

In this work, transmission through planar contaminating material samples on plastic substrates is measured, where the signal model is a two-port network with *S*-parameters, $S_{11}$, $S_{21}$, $S_{12}$, and $S_{22}$. The parameter of interest is the one-way transmission, i.e., $S_{21}$, which is affected by the losses in the material under test (MUT) and by the matching losses between the different materials. Three different measurement setups were used to determine the losses due to different types of contamination: (1) A radome tester with a laterally spatial-resolved measurement of a transmission, (2) a vector network analyzer (VNA) with time-gated measurement of a transmission in a free space [26], and (3) a radar target simulator (RTS) for measurements of the effect of contamination on a complete automotive radar sensor.

### 2.2. Measurement Setup of Radome Tester

To measure and analyze the loss characteristics of various types of contamination, a radome tester (R&S QAR50) [27] was used. This is an instrument specifically developed to measure the attenuation of a car radar signal when it passes through a radome. The radome tester has a structure similar to a VNA in a free-space setup, with built-in calibration routines. However, the radome tester is equipped with a transmitter antenna array and a receiver antenna array, enabling lateral resolution in spatial domain losses in the MUT to be resolved laterally in the spatial dimension.

**Figure 1:**
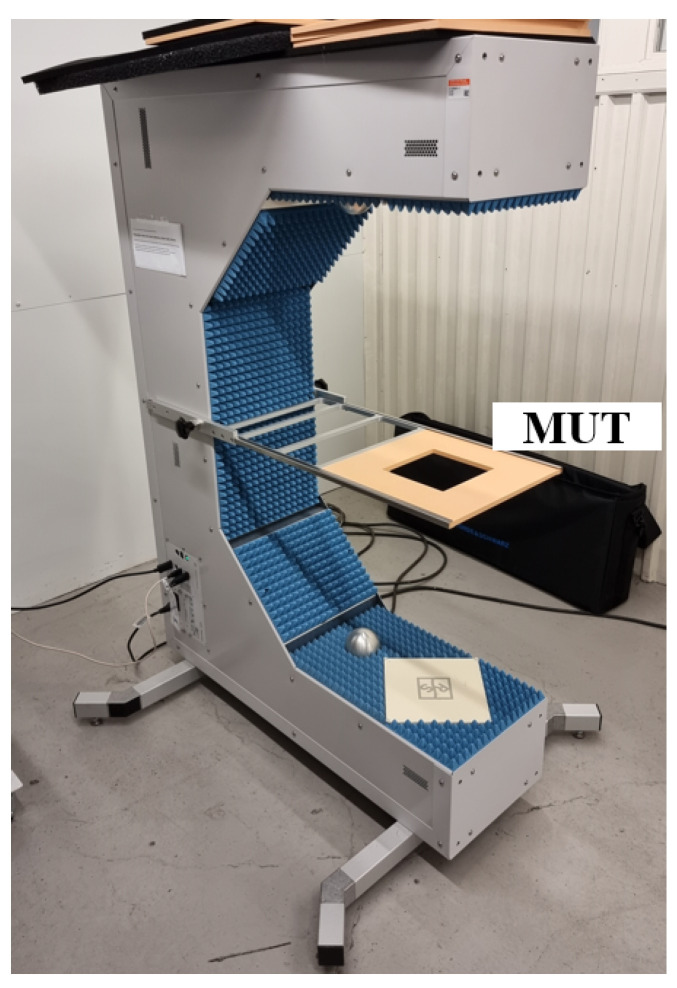
Measurement setup of radome tester.

Figure 1 shows the measurement setup for the radome tester. It consists of two coherent multiple-input multiple-output antenna arrays, a frequency synthesizer, and a graphics processing unit. The antenna array, which is arranged on a square frame, has 94 co-polarized transmit antennas, two cross-polarized transmit antennas, 94 co-polarized receive antennas, and two cross-polarized receive antennas. Therefore, the measurement results can be provided as a 2D image. In this work, the transmission attenuation is measured in the frequency range from 76 GHz to 81 GHz. For transmission measurements, the resulting image is shown as transmission attenuation in dB, which is the average of the receiving cluster and transmitting cluster measurements. MUT is positioned horizontally using a centrally placed fixed frame, which can facilitate the measurement of water and dust. By maintaining the horizontal surface, the materials remain in place, preserving their distribution and shape, which is critical for precise transmission attenuation measurements and ensures repeatable and reliable data.

### 2.3. Measurement Setup of Vector Network Analyzer

A VNA is a general instrument used to measure the *S*-parameters of microwave circuits. It is characterized by a high dynamic measurement range and several sophisticated methods for calibrating the measurement setup. The measurements are made in the frequency domain. When using a VNA for material characterization, electromagnetic model parameters are usually extracted from the measurements. Examples of typical model parameters are permittivity (ϵ), permeability (μ), and conductivity or resistivity (ρ). The measurements can be performed in a free-space setup. It is possible to achieve very good measurement accuracy using measurements of known material samples for calibration. The parameter of the one-way transmission $S_{21}$ can be expressed as follows:

(2) $$S_{21}=\frac{T(1-\Gamma^{2})}{1-\Gamma^{2}T^{2}},$$

where $\Gamma =X\pm \sqrt{X^{2}-1}$ is the reflection coefficient, $T=\frac{S_{11}+S_{21}-\Gamma }{1-(S_{11}+S_{21})\Gamma }$ is the transmission coefficient, $S_{11}=\frac{\Gamma (1-T^{2})}{\left(1-\Gamma^{2}T^{2}\right)}$, and $X=\frac{S_{11}^{2}-S_{21}^{2}+1}{2S_{11}}$.

The goal is to determine the resulting attenuation caused by the MUT rather than extracting any model parameters. A simplified calibration method has been chosen to enable simultaneous testing with the VNA (R&S ZNA43 with ZC90 extenders) [28,29] and the radar target simulator. The interference from multipath propagation is eliminated by filtering out the first incident signal using time gating. This is carried out using the built-in inverse Fourier transform in the VNA to transform the measured signal from the frequency domain to the time domain. The time-filtered signal undergoes a Fourier transform to arrive, again, at frequency-resolved data. The transmission loss through the plastic substrate is measured and subtracted from the measurements of each sample to find the MUT signal attenuation.

The aim is to create a measurement setup where plane wavefronts incident on the MUT. For a perfectly flat sample, this means that no refraction will occur. This is also a well-defined line-up that is possible to recreate. A difficulty with this method is that the MUT may depart from an ideal flat surface and that refraction may either focus or defocus the signal to be measured.

**Figure 2:**
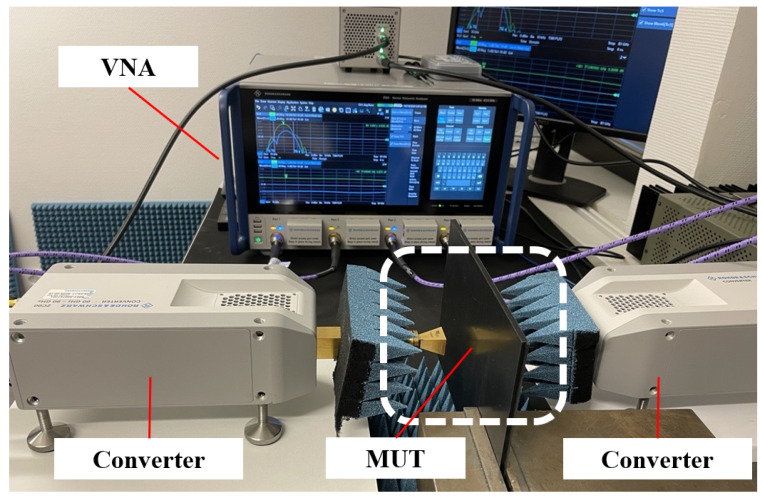
Measurement setup of the vector network analyzer.

Figure 2 shows the VNA measurement setup where all measurements are conducted in the 76 GHz to 81 GHz frequency range. To achieve this, two converters are used. The plate is positioned between two facing antennas, with the transmitter placed 5 mm from the plate and the receiver positioned 25 mm away. This configuration allows accurate and consistent signal transmission by penetrating contamination generated in a limited area on the MUT, allowing for precise evaluation of transmission characteristics over the frequency range.

### 2.4. Measurement Setup of Radar Target Simulator

The actual effect of contamination on a radar sensor is finally tested using an RTS (R&S AREG800A) [30]. The RTS receives the emitted signal from the radar sensor under test; it attenuates the signal and delays and frequency-shifts it to simulate a response from a distant and moving target with a specified radar cross-section.

**Figure 3:**
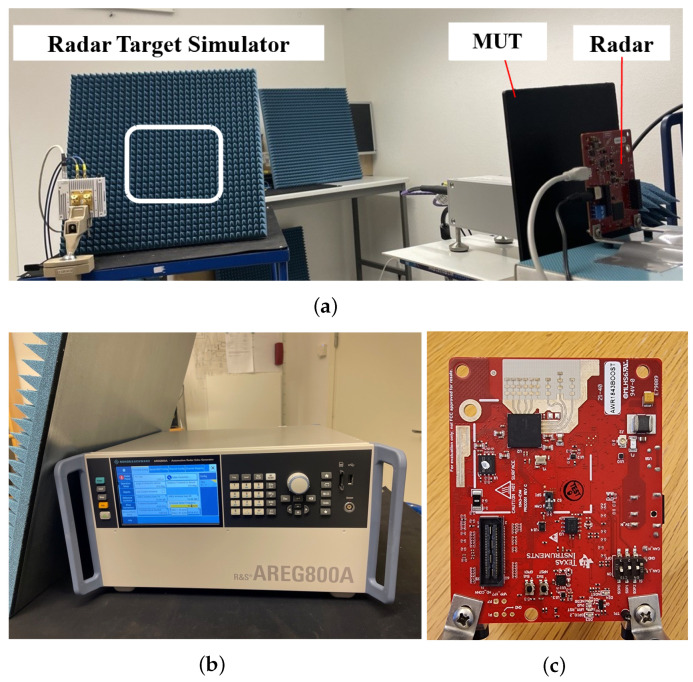
Measurement setup of automotive radar target simulator system. (**a**) Overview of sensor placement. (**b**) Radar target simulator. (**c**) Test radar.

Figure 3 shows the measurement setup using the RTS together with a test radar. The RTS is set to a stationary object with a radar cross-section of 20 dBsm, located at a distance of 30 m. The test radar measures the relative power of the signals returned from the RTS. The test radar is a single-chip 76 GHz to 81 GHz automotive radar sensor evaluation module (Texas Instruments AWR1843BOOST) [31]. In this configuration, the plate is fixed 2 cm in front of the radar to create a contamination area that adequately covers the measurement signal emitted by the radar antenna array. The distance between the radar and the RTS antenna is set to 1.5 m, and the signal detection is measured.

The operational frequency range of the radar is from 76 GHz to 81 GHz; this is close to the operating frequency range of a vector network analyzer (VNA). The radar operates at five distinct starting frequencies within this range, with a valid sweep bandwidth of 219 MHz and an end frequency of +0.31 GHz for each frequency. Additionally, the frequency slope is set to 19.55 MHz/µs, the sampling rate to 11.55 Ms/s, and the number of samples to 128. The maximum detectable range is 70 m, providing a range resolution of 0.68 m. Each radar data measurement session lasts 20 s, with the results averaged to enhance reliability.

In long-range detection mode, where the operational range is set to 70 m, the automotive radar emits strong signals that can lead to numerous noise points being reflected from nearby surfaces. To achieve more accurate measurements of the RTS target, it is essential to strategically arrange adequate absorbers and equipment. This setup minimizes interference from noise reflections, enhancing the clarity and precision of the radar’s detection capabilities. Properly controlling the testing environment is crucial for obtaining reliable results. This setup enables a detailed analysis of the radar’s performance under varying contamination conditions, providing insights into the effects of surface contaminants on radar signal transmission and object detection capabilities.

## 3. Results and Discussion

### 3.1. Measurement Results of Water, Dust, and Mud Using Radome Tester

**Figure 4:**
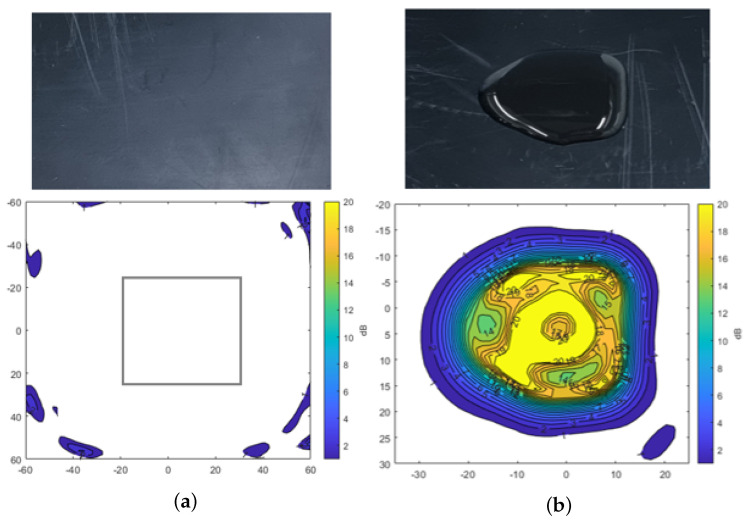
Transmission attenuation measurement result of reference plate and water. (**a**) Reference plate. (**b**) Water.

A radome tester was employed to investigate the impact of water, dust, and mud on radar signal attenuation in com- parison to the uncontaminated plate. The measurements were conducted in static conditions, where the plate was mounted horizontally in the setup. Contaminants were made using demineralized water and Arizona test dust [32] for all measurements. The main components of the Arizona dust include SiO_2_, Al_2_O_3_, Fe_2_O_3_, CaO, and K_2_O. The particle size of A2 fine dust is below 120 µm, while A4 coarse dust is below 200 µm. Figure 4 shows the results of transmission attenuation for the plate after applying 3 mL of water to its surface. The color map in the figure represents the attenuation levels in dB. Figure 4a shows the attenuation measured for the clean plate, where a gray rectangle highlights the evaluation window that is part of the numerical results being evaluated. It indicates minimal transmission loss through the plate. However, Figure 4b shows the considerable signal attenuation exceeding −20 dB in the thick region after water was applied to the plate. This result underscores the significant influence of water on radar signal attenuation.

**Figure 5:**
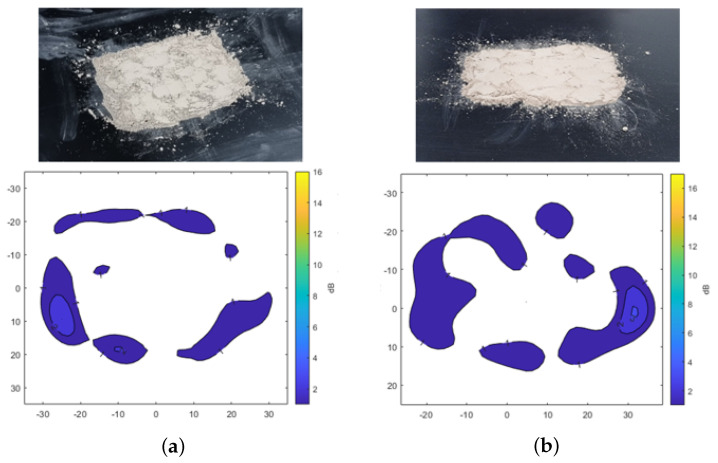
Transmission attenuation measurement results of Arizona test dust. (**a**) A2 fine. (**b**) A4 coarse.

Figure 5 shows the results of transmission attenuation caused by the presence of dust on the plate. In Figure 5a, A2 fine test dust is shown, while Figure 5b presents A4 coarse test dust, both distributed over a 7 cm by 5 cm area, each weighing 3 g. The overall signal attenuation across the dust-covered areas remains relatively low, with a maximum attenuation of −3 dB. Higher attenuation levels are observed only in specific areas where dust has accumulated more densely. This type of heavy accumulation is unlikely to occur on the sensor surface of the vehicle, which is typically positioned vertically. Therefore, the analysis confirms that dust contamination results in lower signal attenuation compared to the significant attenuation caused by the presence of water.

Three different types of mud were created to investigate the effects of mud, a combination of water and dust, as well as the impact of dried mud, on signal attenuation. Each type was made by mixing 3 g of test dust with 1.5 mL of demineralized water. The resulting sticky mud was then applied to the plate to ensure consistent coverage.

**Figure 6:**
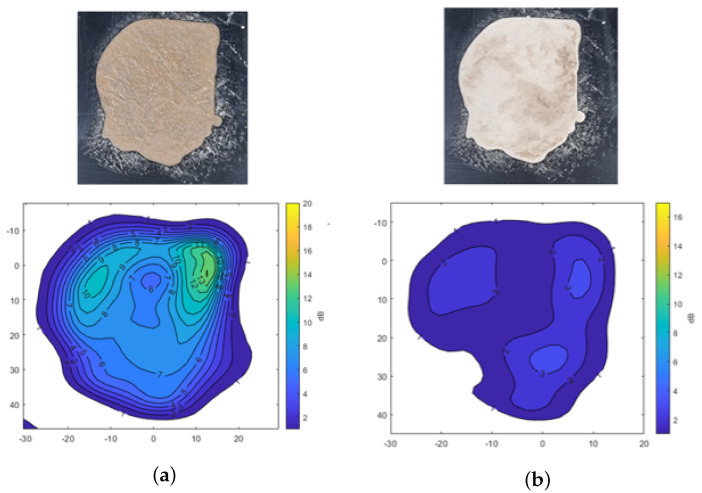
Transmission attenuation measurement results of mud using A2 fine Arizona test dust. (**a**) Mud. (**b**) Dried mud.

Figure 6 shows the transmission attenuation results of mud formed with A2 fine test dust, both in its moist state and after drying. In Figure 6a, signal attenuation is mapped across the surface, showing attenuation levels of −10 dB and −13 dB in areas with higher moisture content. Meanwhile, Figure 6b highlights a specific area with up to −3 dB of attenuation, indicating that even visually dried mud retains moisture, causing residual signal attenuation. This analysis confirms that moisture content significantly affects radar signal transmission, with higher attenuation observed in wetter areas and some attenuation persisting in partially dried mud.

**Figure 7:**
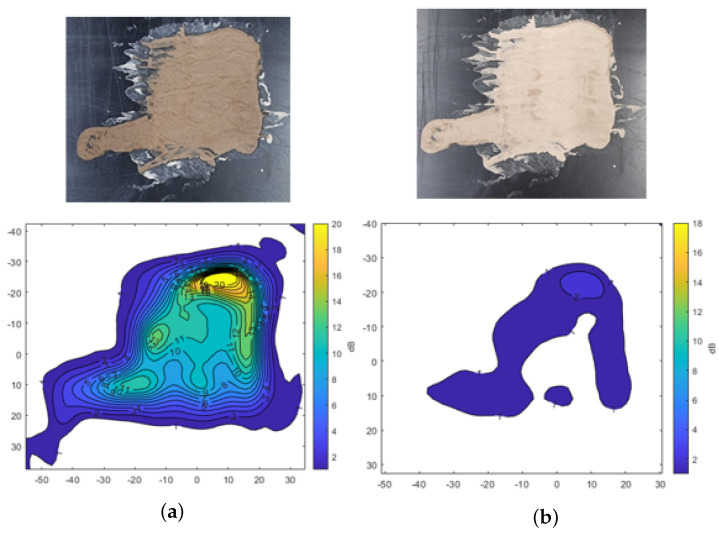
Transmission attenuation measurement results of the mud from A4 coarse Arizona test dust. (**a**) Mud. (**b**) Dried mud.

Figure 7 shows the transmission attenuation results for mud composed of A4 coarse test dust mixed with water. Similar to the findings with A2 fine dust, the transmission attenuation is higher in the initial wet state compared to the dried condition. In Figure 7a, the yellow region represents a thicker mud layer, where attenuation exceeds −20 dB, indicating significant signal loss in areas with substantial moisture. Even in the dried state, shown in Figure 7b, the same region exhibits relatively higher signal attenuation, suggesting that residual moisture in the thicker areas of the mud continues to affect radar signal transmission.

**Figure 8:**
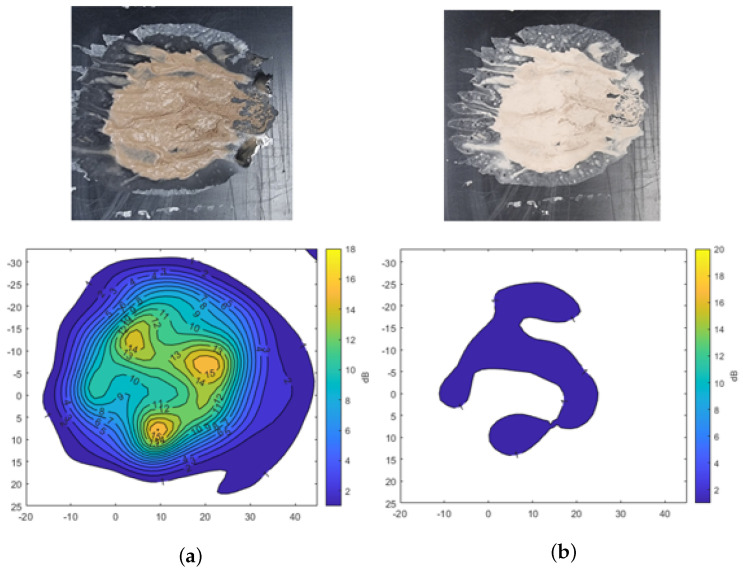
Transmission attenuation measurement results of mixed mud using A2 fine and A4 coarse Arizona test dust. (**a**) Mud. (**b**) Dried mud.

Figure 8 shows the results using mud formed by mixing 1.5 g (each) of A2 fine and A4 coarse test dust with 1.5 mL of water. The irregularly shaped mud spread over the surface produces varying levels of transmission attenuation across the measurement area. The result indicates that signal attenuation reaches up to −15 dB in certain regions, depending on the thickness of the mud. These results also highlight the fact that the thicker areas of the mud layer show more pronounced signal degradation.

The results from the radome tester demonstrate that the presence of water on the sensor surface significantly influences radar signal transmission. Furthermore, mud containing moisture contributes to radar signal attenuation, with the extent of this attenuation dependent on the moisture content. Irregularly shaped mud is particularly noteworthy, as residual moisture tends to remain in specific areas during the drying process, leading to higher signal attenuation in these regions. These findings suggest that it is essential to conduct more quantitative and controlled analyses of the impact of mud on radar signals to better understand and mitigate its effects.

### 3.2. Measurement Results of Moisture and Dust Combination

**Figure 9:**
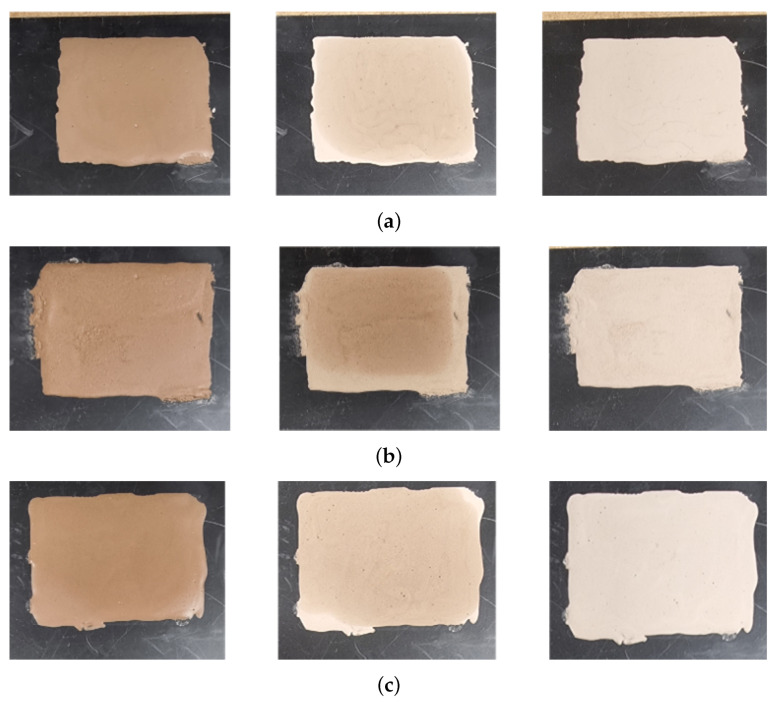
Test mud of different types of dust and moisture. (**a**) Mud composed of A2 fine test dust with demineralized water. (**b**) Mud composed of A4 coarse test dust with demineralized water. (**c**) Mud composed of A2 fine and A4 coarse test dust with demineralized water. Each mud sample was dried and measured under the same conditions.

To achieve a more quantitative analysis of the moisture and dust combination in mud, measurements were conducted under controlled conditions using dust and water. Figure 9 shows the state of the mud samples prepared for measurement under specific conditions. To create the mud, 3 g of test dust and 1.5 mL of demineralized water were mixed. Figure 9a represents mud made with A2 fine dust, Figure 9b with A4 coarse dust, and Figure 9c with a mixture of 1.5 g of each dust type. Each mud sample was applied onto the plate using a mold with dimensions of 6 cm (width), 5 cm (length), and 0.2 cm (thickness). Based on the drying conditions in an oven maintained at 42 °C, the samples were categorized into the following states: initial ‘init’ state (left column), 5-min dried ‘5 min dry’ state (middle column), and 10-min dried ‘10 min dry’ state (right column). Under the test conditions, mud that was dried for 5 min still contained moisture compared to mud that was dried for 10 min.

VNA and RTS measurements were conducted using the mud applied to the plate, as shown in Figure 9. Both methods examined the same contaminated plate conditions but from different perspectives: the VNA measured the transmission parameters of signals passing through the contaminated plate, whereas the RTS measured the relative power of detected objects, simulating an automotive radar scenario. The uncontaminated plate state is labeled “plate” in the figures for reference, and the tests were conducted across the frequency range from 76 GHz to 81 GHz.

#### 3.2.1. Measurement Results Using VNA

**Figure 10:**
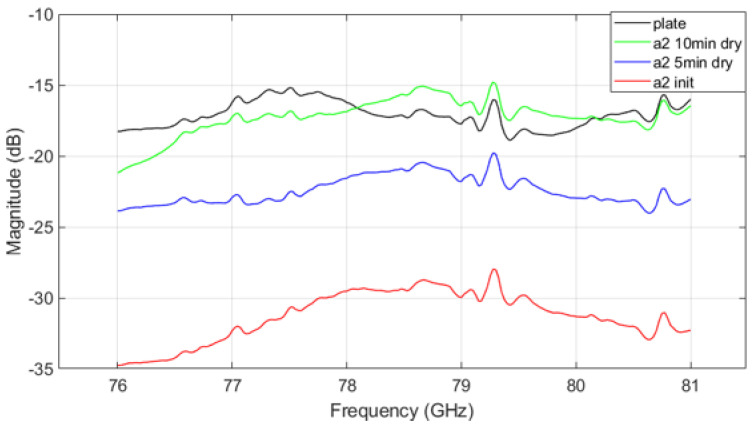
VNA measurement result of A2 fine mud.

**Figure 11:**
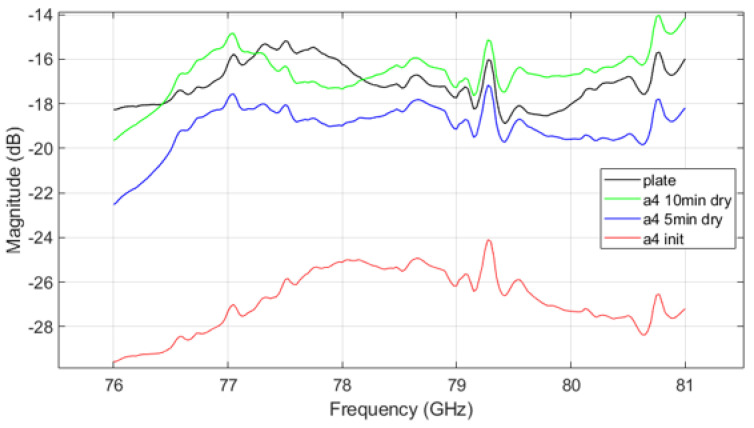
VNA measurement result of A4 coarse mud.

**Figure 12:**
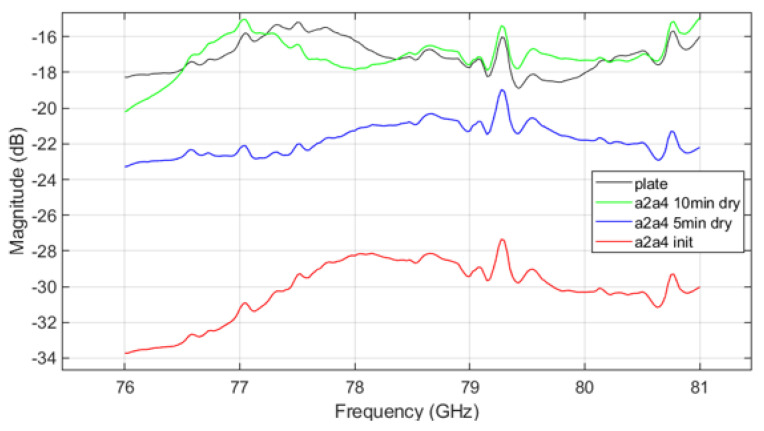
VNA measurement result of A2 fine and A4 coarse mixed mud.

Figure 10, Figure 11 and Figure 12 show the VNA results for different mud types. Figure 10 shows the transmission parameter caused by mud from A2 fine test dust. In the initial state, significant signal attenuation is observed across all frequencies. As the mud dries, signal attenuation decreases, though the mud still retains some moisture after 5 min of drying, which continues to influence signal transmission. A consistent trend across contamination states is that signal attenuation decreases and then increases again around the 79 GHz frequency range, differing from the uncontaminated plate’s parameter graph. This trend highlights the critical role of moisture and drying time on radar signal performance in contaminated environments.

Figure 11 shows the VNA measurement results for mud from A4 coarse test dust, while Figure 12 shows results for mud mixed from both A2 fine and A4 coarse dusts. The trends in parameter measurements over the drying time in the results follow a consistent pattern with those observed in Figure 10. As drying progresses, signal attenuation diminishes, but moisture still affects the transmission characteristics. Signal attenuation is significantly higher in the initial wet state compared to the dried state, but even after drying, the remaining mud on the plate continues to affect signal transmission. This suggests that the transmission pattern of radar signals through the plate contaminated with dried mud differs from that of a clean plate, demonstrating that both the presence of dust and moisture, as well as the drying process, can substantially affect radar signal performance.

Table 1 provides the quantitative results of relative signal attenuation at each frequency. The lowest values for each mud type are highlighted in bold. The initial mud state exhibits the lowest relative signal attenuation for all mud types and conditions. Additionally, the dried mud exhibits signal gain compared to the reference plate at certain relatively high frequencies (79–81 GHz), which is also highlighted.

The VNA measurement results indicate that in the initial ’init’ state, mud containing A2 fine dust exhibits a higher signal loss compared to the other types of mud (see Figure 10 and Figure 12). It is important to acknowledge potential errors that could arise due to variations in temperature within the oven and differences in the timing of repeated measurements. Following each mud test, the contamination on the plate was thoroughly cleaned and allowed to dry completely. Upon re-measurement, the transmission parameter graph returned to resemble that of the uncontaminated ’plate’ state. This suggests that the residual mud from previous measurements continues to influence the transmission characteristics as a function of frequency. Such findings highlight the complex interplay between dust composition and moisture content in determining radar signal behavior, necessitating careful control of experimental conditions to minimize variability. The peaks appearing at 79.3 GHz and 80.7 GHz in Figure 10, Figure 11 and Figure 12 may be inherent resonances in the test plates or an artifact from the calibration. This does not affect the overall conclusion but should be further investigated in future work.

#### 3.2.2. Measurement Results Using RTS and Radar

**Figure 13:**
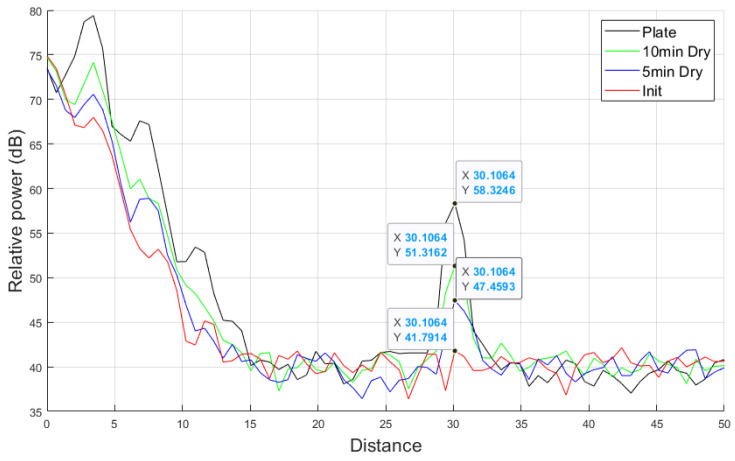
Automotive radar and radar target simulator system measurement result of A2 fine mud at 77 GHz frequency.

Figure 13 shows the object detection performance and relative power results of the automotive radar using RTS in the operational range of 77 GHz with A2 fine test dust mud applied. The relative power decreases with increasing distance, and the system successfully detects the stationary target set at 30 m, confirming that the system operates as intended. The detected object distance of 30.1 m is within the measurement error range. Additionally, compared to the clean plate, which measured a relative power of 58.32 dB, the system recorded 41.79 dB with initial mud, 47.45 dB with 5 min dried mud, and 51.31 dB with 10 min dried mud. The initial mud state causes greater signal attenuation, indicating its impact on target detection based on the relative power distribution within the target distance range. These results indicate that mud can degrade the object detection performance of the automotive radar.

**Figure 14:**
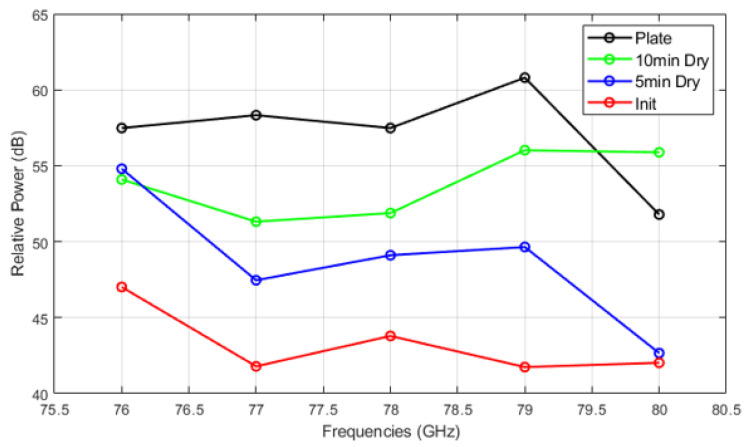
Relative power of automotive radar for target detected from radar target simulator (A2 fine mud).

**Figure 15:**
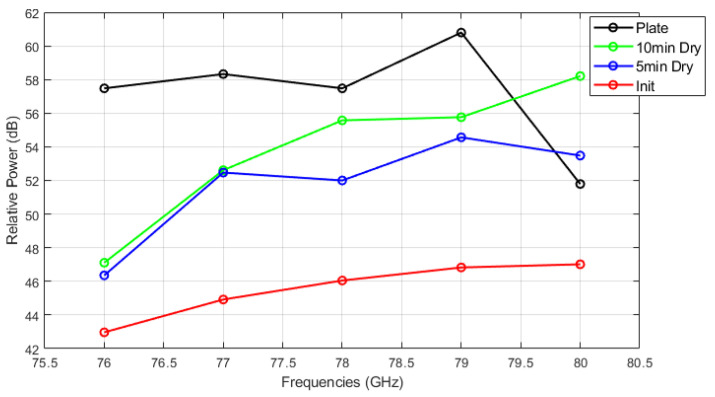
Relative power of automotive radar for target detected from radar target simulator (A4 coarse mud).

**Figure 16:**
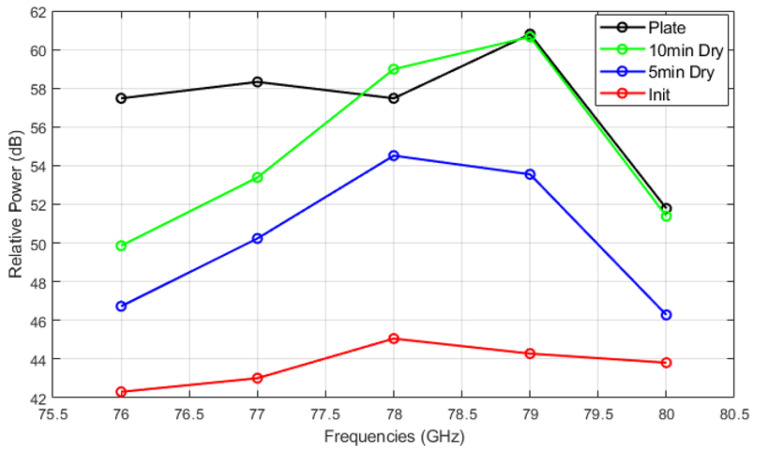
Relative power of automotive radar for target detected from radar target simulator (A2 fine and A4 coarse mixed mud).

Figure 14, Figure 15 and Figure 16 show the relative power measurements of detected objects within each radar frequency range under three types of mud contamination and their drying conditions. The results reveal that the presence of mud with high moisture content in its initial state greatly diminishes the radar’s ability to detect objects by significantly lowering the relative power. In some cases, such as Figure 13, this can even lead to detection failure. As the mud dries and moisture evaporates, the radar’s detection capabilities improve, as seen by the relative power peaks that align with distance measurements. However, residual contaminants on the sensor surface continue to cause signal attenuation, particularly at specific frequencies such as 76 GHz and 77 GHz, even after drying.

The quantitative results of relative power at each frequency are presented in Table 2. The lowest values for each mud type are highlighted in bold. Similar to the VNA results, the initial mud state exhibits the lowest relative power for all mud types and conditions, indicating that the water content in the initial mud causes significant signal attenuation.

The results of three measurements indicate that the moisture content in mud significantly impacts the radar signal and its ability to detect objects, with higher moisture leading to greater attenuation of the signal. Higher-density materials can also be expected to have greater signal losses compared to materials with air mixed in. Powder materials can, when mixed with water, change in density after sedimentation and drying. However, the combined influence of A2 fine and A4 coarse dust on object detection performance remains inconclusive in this study, suggesting that the relationship between the dust types and radar signal attenuation requires further exploration. Future research should focus on conducting more controlled and quantitative experiments, incorporating a wider range of contaminants, and assessing the radar’s detection capabilities under various conditions. This will provide a clearer understanding of how different types of surface contamination affect automotive radar performance, allowing for better insights into mitigating signal loss in real-world scenarios.

## 4. Conclusions

This study helps quantify the impact of environmental contaminants, specifically water, dust, and mud, on the performance of radar sensors operating at frequencies used in AD/ADAS. The experiments conducted with a 76–81 GHz radar sensor reveal that moisture can lead to substantial signal attenuation, thus reducing the effective detection range of the radar by half. In contrast, dust contamination resulted in comparatively lower attenuation levels (up to −3 dB), reducing the effective detection range by 10%), reinforcing the notion that the type and amount of contaminant play a crucial role in sensor performance. Moreover, the dynamic behavior of mud, comprising both water and dust, further complicates radar operation. The moist mud contaminant exhibited notable signal degradation, with moisture content proving to be a pivotal factor in determining attenuation levels (up to −20 dB). The persistence of signal loss in areas of dried mud underscores the importance of designing effective moisture management solutions for radar systems. The experimental results provided in this study serve as a critical foundation for predicting radar performance in real-world automotive environments. The results emphasize the necessity for robust sensor surface design and maintenance protocols in the context of AD/ADAS and clear efforts at the vehicle design stage to mitigate contaminant build-up at the location of radar sensor radomes. Future research will explore utilizing these lab studies in environmental wind tunnel environments to help transfer laboratory results to more complex test environments.

## Figures and Tables

**Table 1:** Relative signal attenuation (dB) under different conditions for various mud types.

Mud Type	Conditions	Relative Attenuation at Each Frequency (dB)					
		76 Hz	77 Hz	78 Hz	79 Hz	80 Hz	81 Hz
A2 fine mud	Init	**−16.50**	**−16.09**	**−13.29**	**−12.23**	**−13.28**	**−16.28**
	5 min Dry	−5.61	−6.70	−5.33	−4.06	−4.93	−7.05
	10 min Dry	−2.91	−1.02	−0.64	**1.26**	**0.68**	−0.46
A4 coarse mud	Init	**−11.30**	**−11.1**	**−8.88**	**−8.44**	**−9.28**	**−11.21**
	5 min Dry	−4.26	−1.57	−2.75	−1.41	−1.56	−2.20
	10 min Dry	−1.38	1.17	−1.10	**0.45**	**1.30**	**1.82**
A2&A4 mixed mud	Init	**−15.46**	**−15.02**	**−12.03**	**−11.69**	**−12.28**	**−14.03**
	5 min Dry	−5.01	−6.04	−5.03	−3.55	−3.76	−6.20
	10 min Dry	−1.93	**0.99**	−1.54	**0.15**	**0.71**	**1.05**

**Table 2:** Relative power measurements under different conditions for various mud types.

Mud Type	Conditions	Relative Power at Each Frequency (dB)				
		76 Hz	77 Hz	78 Hz	79 Hz	80 Hz
A2 fine mud	Plate	57.4779	58.3246	57.4779	60.7939	51.7866
	Init	**47.0124**	**41.7914**	**43.7905**	**41.7444**	**42.0266**
	5 min Dry	54.7969	47.4593	49.1055	49.6464	42.6616
	10 min Dry	54.0913	51.3162	51.8806	56.0198	55.8787
A4 coarse mud	Plate	57.4779	58.3246	57.4779	60.7939	51.7866
	Init	**42.9673**	**44.9193**	**46.0482**	**46.8243**	**47.0124**
	5 min Dry	46.3539	52.4686	51.9982	54.5617	53.4799
	10 min Dry	47.1065	52.6097	55.5730	55.7611	58.2070
A2&A4 mixed mud	Plate	57.4779	58.3246	57.4779	60.7939	51.7866
	Init	**42.3088**	**43.0144**	**45.0604**	**44.2843**	**43.814**
	5 min Dry	46.7302	50.2344	54.5147	53.5504	46.2834
	10 min Dry	49.8581	53.3858	58.9831	60.6528	51.3868

## Data Availability

Data are contained within the article.

## Abbreviations

The following abbreviations are used in this manuscript:

AD	Autonomous driving
ADAS	Advanced Driver Assistance Systems
MUT	Material under test
RTS	Radar target simulator
VNA	Vector network analyzer
